# Knowing Is Not Enough: A Mixed-Methods Study of Antimicrobial Resistance Knowledge, Attitudes, and Practises Among Maasai Pastoralists

**DOI:** 10.3389/fvets.2021.645851

**Published:** 2021-03-22

**Authors:** Peter E. Mangesho, Mark A. Caudell, Elibariki R. Mwakapeje, Moses Ole-Neselle, Tabitha Kimani, Alejandro Dorado-García, Emmanuel Kabali, Folorunso O. Fasina

**Affiliations:** ^1^Amani Medical Research Centre, National Institute for Medical Research, Muheza, Tanzania; ^2^Food and Agriculture Organization of the United Nations, United Nations Complex, Nairobi, Kenya; ^3^Food and Agriculture Organization of the United Nations, Dar es Salaam, Tanzania; ^4^Food and Agriculture Organization of the United Nations, Rome, Italy; ^5^Woodham La, Addlestone, United Kingdom

**Keywords:** antimicrobial stewardship, knowledge attitudes and practises, antimicrobial use, antimicrobial resistance, Maasai, Tanzania

## Abstract

Global, national, and local efforts to limit antimicrobial resistance (AMR) often stress the importance of raising awareness among users, sellers, and prescribers of antimicrobial drugs. This emphasis is founded upon two assumptions. First, awareness is limited, particularly concerning the links between antimicrobial use (AMU) and AMR. Second, “filling the awareness gaps” will motivate practises that will limit AMR. The first assumption is supported by knowledge, attitudes, and practises (KAP) surveys but these same studies provide mixed support for the second, with several studies finding that knowledge and attitudes are not correlated with related practises. This disconnect may arise as these surveys typically do not collect data on the cultural or historical contexts that pattern AMU. To explore how these contexts impact KAP related to AMU and AMR, we use a mixed-methods approach to examine veterinary practises among Maasai pastoralists in Tanzania. We combine a quantitative KAP survey (*N* = 195 households) with extensive qualitative data from focus group discussions (*N* = 55 participants). Results document limited awareness of AMR but also find that knowledge and attitudes are not correlated with practise. Thematic analysis of qualitative data pointed to three reasons behind this disconnect, including (1) Maasai self-perceptions as veterinary experts, (2) the central role of livestock in Maasai culture, and (3) the use of ethnoveterinary knowledge in animal health treatment. We argue that mixed-method approaches will be critical to developing the targeted awareness campaigns needed to limit the emergence and transmission of AMR.

## Introduction

The health and economic threat posed by antimicrobial resistance (AMR) globally has motivated international agencies, including the WHO and the Food and Agriculture of the United Nations (FAO) to develop and implement global action plans and strategies to address AMR (1–3). These global plans, which are often emulated at national levels, place a strong emphasis on improving AMR awareness. This emphasis is driven by two assumptions. First, gaps in awareness of AMR exist in public health and agricultural sectors and these gaps give rise to poor practises. Of these practises, particular focus has been placed on the misuse of antimicrobials (e.g., not taking a full dose) as misuse is believed to be the major selective force patterning AMR in humans, animals, and the environment (4). Second, it is assumed that by “filling in” these awareness gaps that users, prescribers, and sellers of antimicrobials will engage in more prudent antimicrobial use (AMU) and related practises, thereby limiting the emergence and transmission of AMR. The global embodiment of these assumptions is the WHO's World Antibiotic Awareness Week (WAAW), with its central objectives “to improve awareness and understanding of AMR through effective communication, education and training” in order to “encourage best practises among the general public, health workers, and policymakers” (5).

The assumptions supporting AMR awareness efforts can be assessed through results of knowledge, attitudes, and practises (KAP) surveys (6–13). KAP surveys are designed to quantify the gaps in AMR knowledge (e.g., can you explain AMR?), attitudes, (e.g., do you believe that AMR will impact your livelihood?), and practises (e.g., do you always take a full dose of antibiotics?). KAP surveys are particularly relevant for addressing AMR in low- and middle-income settings (LMICs). This relevance, in part, stems from limitations in the enforcement of AMR relevant policies (e.g., prescription laws) and public and animal health services common with LMICs (6). Due to these limitations, AMU and AMR-relevant decisions are often made at the level of individual users and their social networks (14). Given these decisions occur at the user level, understanding the KAP of these users is critical to developing targeted awareness campaigns in LMICs.

Results of KAP studies provide support for the first assumption behind awareness-raising campaigns—that gaps exist in awareness and knowledge of AMR and prudent AMU—but provide more mixed support for the second assumption—that increases in awareness will promote behaviour change. In terms of the former, KAP surveys across public health and agricultural sectors find that awareness of AMR is often limited and non-prudent practises common, particularly in LMICs where antimicrobials are often bought over the counter without a prescription (6–13). However, in questioning the second assumption, these studies also find that awareness levels are not significantly associated with practises at the individual level (6, 9, 15). That is, those who are most aware of AMR and/or hold the most prudent attitudes are no more likely to observe prudent AMU practises. This lack of association has been documented in farmers and sellers of antimicrobials in Asia and Africa and in both public and animal health settings (6, 9).

Although limited in number, results of KAP studies suggest that awareness is not the primary driver of practises related to AMU and AMR. This disconnect between awareness and behaviour has been well-documented in areas across public health and is driven by factors that lie both within and outside the individual (16, 17). Psychological biases, such as a preference for short-term rewards limit the observance of practises whose benefits might only be realised in the long-term (e.g., reduce AMR from prudent AMU) (18). Factors outside the individual, such as workforce infrastructures for public and animal health and cultural norms of health also determine options available and so the likelihood of acting on awareness gains (e.g., finding a veterinarian to get a prescription for antibiotics) (14, 17, 19–21). These factors, especially those structural reasons that exist outside of the individual, are rarely assessed in KAP studies (22, 23). To investigate how these factors interact with KAP related to AMU and AMR, as well as their correlation, we conducted a mixed-methods ethnographic study of animal healthcare practises among Maasai pastoralists in northern Tanzania.

## Methods

### Study Population

The Maasai live throughout northern Tanzania and southern Kenya and continue to pursue a pastoralist livelihood. Due to different pressures on their grazing lands, including neighbouring farming communities converting rangeland into agricultural fields, most Maasai are diversifying their livelihoods by growing some food crops and venturing into the tourism industry, mining, and operating guesthouses (24, 25). Most Maasai inhabit rural areas within extended family compounds called *bomas* (*enkang* in *Maa*) organised along patrilineal lines. Polygamy remains common, especially in rural areas, and marriages are structured by patrilocal residence and clan exogamy. Bride price, usually paid in livestock, is still required. Livestock is grazed over a large area during the daytime and brought back home in the evening where they are kept inside the *boma* with smallstock (sheep and goats) and large stock (cattle) kept separately. Livestock remains important for the Maasai, both economically and culturally, and livestock care begins at a young age within Maasai communities. Children begin tending livestock as early as 5 or 6 years, initially by tending kids/calves, which stay around the *boma*. Boys progress to herding adult goats, sheep, and cattle as they age and become *moran* (warriors) (26–28) while girls and women may tend small stock but also begin to focus on household chores and child care. Older Maasai men spend their time tending to livestock mainly through overseeing the young herders and in business ventures, including work in Tanzanite mines, operating guesthouses, and as security guards at banks and safari companies (29–31).

### Study Design and Sampling

**Figure 1:**
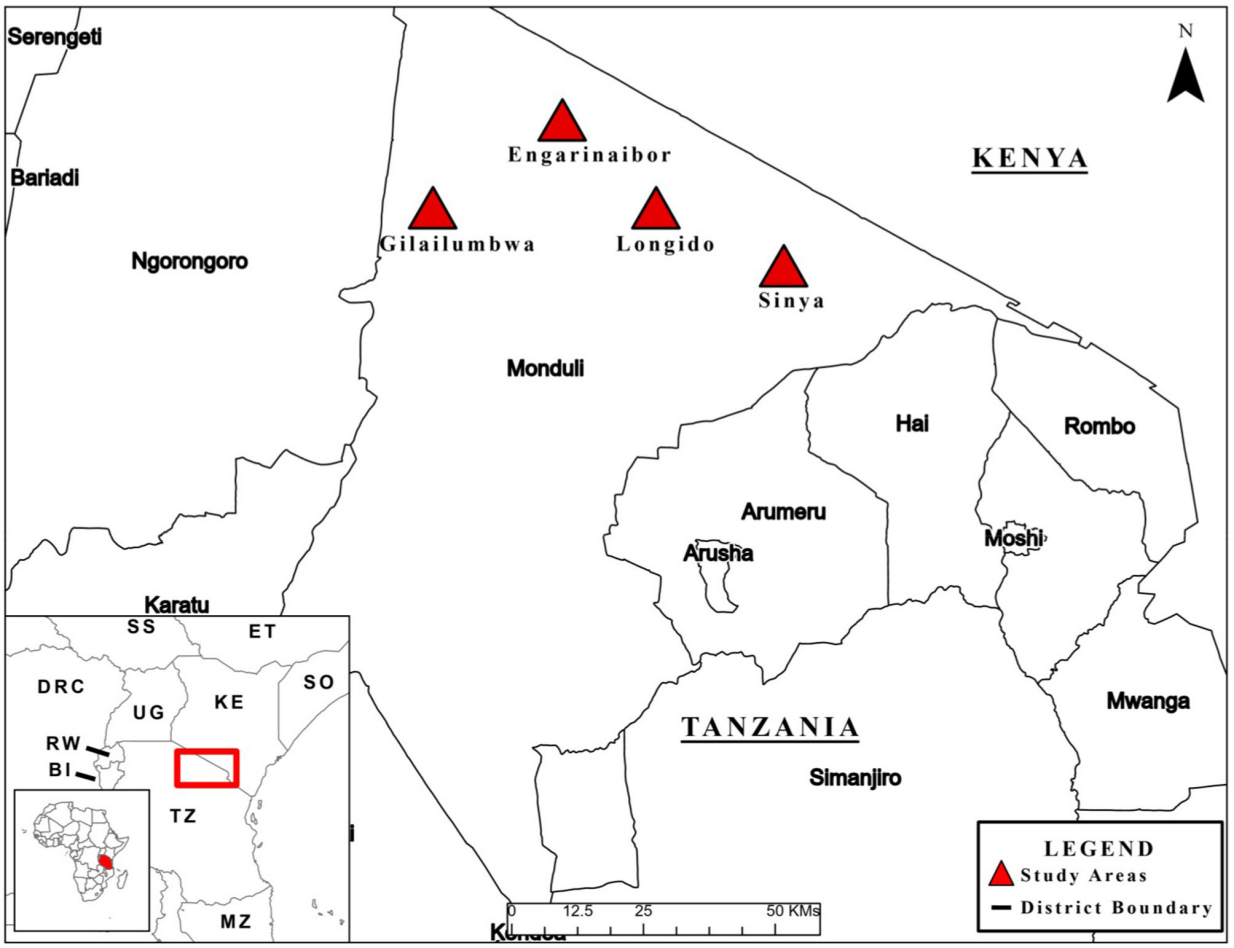
Map of study area. See map legend for description of map markers. First published in (6).

The mixed-methods study, informed by the exploratory sequential study design (32), was carried out in Longido District, Arusha Region in northern Tanzania between November 14th and December 14th 2018 (see Figure 1). Four wards within Longido District were targeted for sampling, including: (1) Orbomba, (2) Isinya, (3) Gilailumbwa, and (4) Engarenaibor. Participants in qualitative interviews, including focus group discussions (FGDs), were purposively selected. Selected individuals came from one village within each of these four locations. In each FGD group, participants were selected came from all hamlets within a village to ensure some form of representation of village views. For the quantitative KAP survey, two purposive selection strategies were used including visiting Maasai marketplaces and Maasai *bomas*. Given we used purposive sampling, KAP results should not be considered representative of the Maasai generally or Maasai living within the four wards. See Supplementary Material, section Study Locations for more details on the selection of wards and sampling for the KAP survey.

### Qualitative Data Themes and Data Analysis

Qualitative data were collected on themes related to AMU and AMR including farm management and economic practises, livestock disease histories, and KAP relating to AMU and AMR, including use, governance, regulations, policies, and enforcement. FGDs were conducted with livestock keepers, who were mostly heads of households, both male and female. See Supplementary Material, Section Qualitative Themes and Tool Development for a list of FGD prompts based upon these themes.

Qualitative data analysis was guided by the content thematic analysis approach (33, 34). Qualitative data analysis began with transcribing audio recordings from FGDs, which were a mixture of *Kiswahili* and *Maa* (i.e., language of the Maasai,). After transcription into text, documents were translated to English following a meaning-based translation approach (35). Initial data analysis was conducted by coding data guided by a deductive framework approach whereby data was coded to the pre-designed categorical themes (33, 34). From the main themes, emerging sub-themes were further coded and collated into potential themes or merged to the main ones. The coding activity was input to and organised with the assistance of QSR Nvivo 8 (QSR International Pty Ltd 2009) software.

### Quantitative Survey and Data Analysis

Thematic analysis was used to develop and refine a quantitative KAP survey tool. KAP questionnaires were administered by local Maasai enumerators using the Kobo Collect^®^ application. Surveys were piloted prior to administration to ensure question clarity and conduct further refinement. KAP surveys took between 45 min and 1 h to complete. Data cleaning, coding, and analysis were completed in Stata 16.1 (36). To assess the correlation between KAP Fisher's exact tests were used given the binary nature of the data and as some cells had fewer than five observations.

### Ethical Approval

The study was reviewed and approved by the Medical Research Coordinating Committee of the National Institute for Medical Research (NIMR) in Tanzania and certificate clearance no. NIMR/HQ/R.8a/Vol.IX/2926 was issued. Prior to requesting for consent, an information sheet containing a detailed narrative of the study and its aims was provided to potential participants. Participants were informed of the benefits and risks of participation and assured of their right to withdraw from study participation at any point. Written informed consent was sought from all study participants who could write. For those who could not write, a thumbprint signature was requested.

## Results

### Descriptive Statistics

#### Demographic Data From FGDs

A total of six FGDs were held with 55 Maasai participants. A slight majority of participants were 33 males (60%) vs. 22 (40%) females. Most participants were married 47 (84%) with a majority (53%) falling into the 41–80 age bracket. About half of participants had no education 27 (48%) with fewer having a primary education 25 (45%) and only three participants (5%) having a secondary education. See Supplementary Tables 1, 2 for more demographic details on FGD participants.

#### Demographic, Socioeconomic, and AMU Data From KAP Surveys

A total of 195 Maasai were interviewed for the KAP survey. Most participants were male 182 (93%), with a majority 120 (62%) having no education, 58 (30%) having some/completed primary, 13 (7%) some/completed secondary, and 4 (2%) some/completed college. The average household was composed of 8–9 people and owned an average of 146 cattle and 230 small stock (sheep and goats), three donkeys, and three chickens. Households cultivated an average of 18 hectares of farmed land. Twelve percent (23) of households had electricity while 124 (64%) households had pit toilets with the remaining using open defecation. Across the 195 households, 21 different drugs were reported to be used with 36% being antibiotics. Sixty-five percent of antibiotics were tetracycline-based drugs, while 28% were penicillin-streptomycin combinations, and 7% were azithromycin-based drugs [see (14) for more details on veterinary drugs used]. See Table 1 for detailed descriptive statistics.

**Table 1:** Demographic and socioeconomic information of Maasai who responded to a knowledge, attitudes, and practises (KAP) survey.

**Variable**	**Mean**	**Sd**	**Min**	**Max**
Age	47.77	13.87	23.00	83.00
Gender (1 = Female, 0 = Male)	0.07	0.25	0.00	1.00
no education	0.61	-	-	-
Primary education	0.30	-	-	-
Secondary education	0.07	-	-	-
College education	0.02	-	-	-
Read (1 = Yes, 0 = No)	0.78	0.41	0.00	1.00
Number of children	8.85	8.73	0.00	50.00
Cattle	146.24	239.97	0.00	1600.00
Small stock (sheep and goats)	230.18	449.76	4.00	5405.00
Chickens	3.34	12.74	0.00	150.00
Donkeys	3.72	6.60	0.00	80.00
Land farmed (hectares)	17.89	68.85	0.00	600.00
Electricity (1 = Yes, 0 = No)	0.12	0.32	0.00	1.00
Flush/Pit toilet (1 = Yes, 0 = No)	0.64	0.48	0.00	1.00

*N = 195 households. The means of binary variables can be interpreted as the percentages of respondents reporting “yes.” For example, around 60% of survey respondents indicated they had no education. “Sd” refers to standard deviation. See Methods section in the main manuscript for a description of survey development and implementation*.

#### Antimicrobial Use and AMR Related Knowledge Measures From KAP Surveys

**Figure 2:**
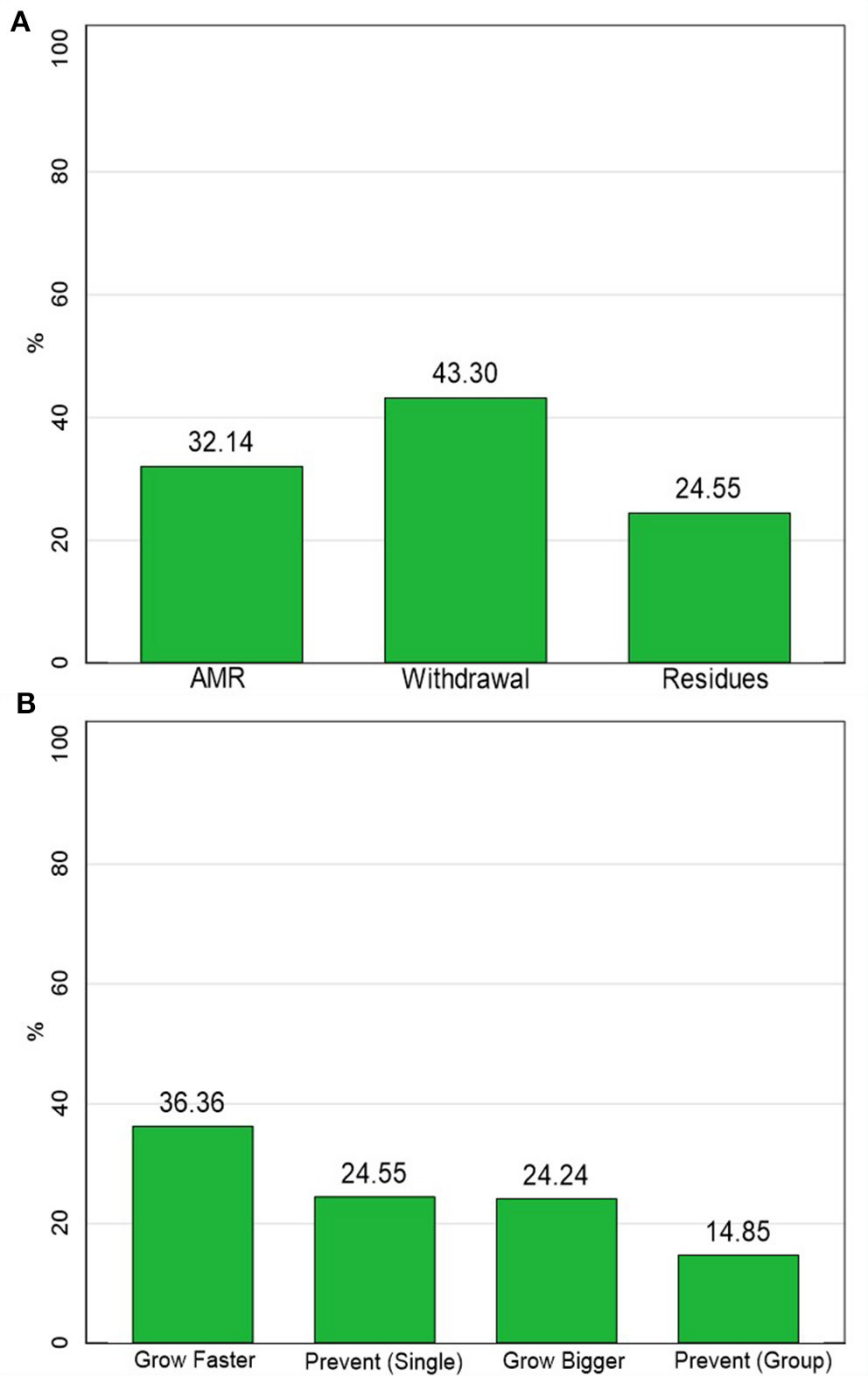
Knowledge measures. **(A)** The percentage of respondents who were aware of AMR-related concepts. **(B)** The percentage of respondents who believed antimicrobials performed growth or prevention functions. *N* = 195.

Less than a third of Maasai had heard of AMR and antibiotic residues while slightly more had heard of withdrawal periods from antimicrobials (see Figure 2). About a third of Maasai said that antimicrobials could help animals grow faster with a quarter believing that antimicrobials prevented animals from getting sick in the future and helped animals grow bigger. Only about 15% thought that giving antimicrobials to an entire group/herd prevented sickness.

#### Antimicrobial Use and AMR Related Attitudinal Measures From KAP Surveys

Most Maasai agreed that treatment with antimicrobials could be stopped when symptoms improved and that giving antimicrobials to healthy animals could help prevent future disease and help them grow bigger and faster (see Table 2). Most Maasai also believed that using antimicrobials inappropriately could limit their effectiveness and that animal health professionals should be consulted before administering antimicrobials. About half of respondents believed that withdrawal periods from animal products (meat and milk) should be observed.

**Table 2:** Agreement across antimicrobial resistance (AMR) attitudinal statements.

**Agreement with the following statements**	**Disagree % (*N*)**	**Neither Agree/Disagree % (*N*)**	**Agree % (*N*)**
You should stop giving AMs if animal improves	9.74 (19)	7.18 (14)	83.08 (162)
Using AMs incorrectly can limit effectiveness	15.38 (30)	11.28 (22)	73.33 (143)
Giving healthy animals AMs will prevent disease	7.28 (14)	9.74 (19)	83.08 (162)
AMs help healthy animals grow bigger and faster	2.05 (4)	15.38 (30)	82.56 (161)
Before administering AMs, you should get consultation from an animal health professional	10.99 (20)	16.48 (30)	72.53 (132)
After using AMs, you should observe the withdrawal period	41.54 (81)	12.31 (24)	46.15 (90)

*AM is antimicrobial. N = 195*.

#### Antimicrobial Use and AMR Related Practise Measures From KAP Surveys

Maasai respondents reported to engage in a variety of non-prudent practises involving drug administration and withdrawal. For treatment length, almost a majority of Maasai 50% (97) said they stop treatment when the animal looks better. Even fewer responded that they consult an animal health professional 19% (37) or the bottle/sachet 17% (36) before administering antimicrobials. Fifty-two percent (101) said they “always” gave drugs to all animals when one showed signs of sickness, while 36% (71) “sometimes” did this and 12% (23%) “never” used group treatment. Overdosing was more common than underdosing with about 36% (70) reporting to use larger than recommended doses “almost always” or “sometimes” and 20% (39%) reporting to underdose “almost always or sometimes.” Almost all respondents (94%, 184) reported to “never/rarely” get a prescription before purchasing antimicrobials. Likewise, almost all the Maasai 85% (166), reported consuming products (meat and milk) from animals within the withdrawal period, with fewer respondents giving products to companion animals 8% (16) or selling the products [2% (3)]. Only 5% (10) Maasai respondents reported throwing away milk and meat products during the withdrawal period.

### Analysis

#### Correlation Between Self-Reported AMU and AMR-Related KAP

Fisher's exact tests showed that most AMU and AMR-related practises were not significantly associated with related knowledge or agreement measures (see Supplementary Table 3 for results of Fisher's exact tests). There were a limited number of exceptions to this trend although some relationships were in unpredicted directions. For example, respondents who agreed that incorrectly using medicines could decrease their effectiveness were significantly more likely (*p* = 0.012) to *not use* the information on bottle/sachet labels or advice from animal health professionals for administration instructions (79.67%) compared to those who *did use* this information and advice (62.50%). Likewise, those who agreed using antimicrobials inappropriately could limit effectiveness were more likely to report treating an entire group when one animal falls ill (94.04%) compared to those who never or rarely reported engaging in this practise (63.36%). More consistent with expectations was that respondents who knew about AMR were significantly less likely (*p* = 0.009) to underdose (68%) compared to those who did not know about AMR (43%). Likewise, those who knew about withdrawal were significantly more likely (*p* = 0.00001) to report observing withdrawal.

### Qualitative Insights on the Gaps Between KAP

#### Lack of Association Between Knowledge and Attitudes Towards AMR and AMU and Dosage/Treatment Practises

Maasai respondents who agreed that inappropriate dosage and treatment courses limited drug effectiveness were no more likely to get advice from veterinarians or take instructions from the bottles or overdose or underdose. Analysis of our FGD data suggested that this lack of association was partially driven by varying definitions of “correct dose.” Among the Maasai, “correctness” is not necessarily something that is associated with the advice of veterinarians or information on the back of a bottle, even though many FGD participants were able to recall the correct dosages of certain antimicrobials from memory. Instead, notions of correctness are based upon long-standing ethnoveterinary norms among the Maasai. In particular, the correct dosage level is judged considering the health of an animal. A healthy-looking animal is perceived as being in a better position to withstand a higher amount of CCs than a weaker more emaciated one. As one respondent said: “*We do not consider the weight, we observe its health status. If it has good health, even if you overdose there is no problem. But if it is somehow weak, you cannot.”* Considerations of weight and health interact to pattern dosage practises, which often produce variable administration practises. As one Maasai man said, “*inject 5 CCs, if I am not mistaken, but if the cattle are healthy. If the cattle's health is poor/deteriorated, you inject 2 CCs or 1 CC if it is a goat, but if the goat is a bit healthy, you inject 1.5 CC. For cattle, you inject 3 CC if its health is not so strong*. R4-FGD-01-MALE_FU-LONGIDO. Conceptions of corrections were also impacted by Maasai ethnoveterinary beliefs concerning the signs of drug-efficacy, especially evidence of its immediate and observable impact on the animal. A dosage level that resulted in animals screaming and/or jumping around is believed to be an indicator of the correct dose, an indicator that the drug was not counterfeit, and evidence that the drug provided the animal with “energy.” We believe this concept of drugs giving animals “energy” is also why many Maasai (i.e., 84%) responded that they use antimicrobials as growth promoters, which contrasts with other studies on pastoralists (38).

#### Lack of Association Between Knowledge and Attitudes Towards Withdrawal and Withdrawal Related Practises

Our FGD data suggested that the lack of association between the observance of withdrawal practises and knowledge and attitudes towards withdrawal can be explained as a product of the role that livestock continues to play in the Maasai culture. While most Maasai respondents acknowledged hearing about withdrawal, a larger majority did not see a problem with consuming an animal that had been recently injected and died. Consumption of already dead livestock without observing prescribed slaughter bylaws is not a problem for Maasai meat handling customs and behaviours. Letting livestock products go to waste was considered “a crime” and a waste of a vital food source. As one Maasai FGD respondent said, “*God gave us the cattle and he cannot allow it to harm us and we cannot destroy them.”* This faith in the purity of livestock was further detailed by an FGD participant:

R2: *Us pastoralists, we do not have that belief in treatment instructions…We consume the products the same day after injection. We are guided by a faith that no matter how and what you injected the animal with, and it died we must eat the meat the same day. The faith we have is that it removes fear or worries on any possible side effects that meat would have posed, we do not fear and we do not get sick* [after consumption]

R2-FGD-MEN-LONG4-004

Another reason for not observing withdrawal was more historical and based upon personal experience. Participants narrated/explained to us that, for many years, they have been consuming products from animals undergoing treatment and dead animals that died unexpectedly after receiving heavy antimicrobial dosages. Despite these practises, they could not recount any negative side effects. In part, this was because as self-described “veterinary experts,” they could tell when meat or milk was unfit for consumption. As one FGD participant told us, again referencing the crime of wasting livestock products:

R6 *For us, there is no meat that we do not consume regardless of the disease that killed that animal, we just prune off the most affected parts, you inspect you throw away that part that you see with your eyes has gone seriously bad but not the whole meat, I am not ready to throw away all the meat, it is a crime…*

FGD-MEN-LONG4-004

A minority of FGD participants did acknowledge that some Maasai are beginning to observe withdrawal, but only for milk and milk products. However, practises surrounding withdrawal were still informed by Maasai beliefs on the drug administration process. Contrary to withdrawal instructions for most antimicrobials, participants reported that milk was safe to drink if held overnight or if the milk was boiled. To the Maasai, boiling rendered milk safe to drink even if the animal had been given drugs minutes/hours before. Maasai responses suggested that boiling both killed diseases and rid the milk of drug residues, the latter belief in contrast with studies showing boiling milk during the withdrawal period does not decrease residue levels (37, 39). Evidence of this conflation was provided by a Maasai respondent, who again framed observance of withdrawal in terms of fear and faith: *Fear is not our culture… but we are slowly changing because we no longer use milk directly from the udder as before, we now boil to kill the bacteria and we believe when you boil the milk from a cow that has been injected with antimicrobials, we believe it is also safe for drinking*.

R2-FGD-MEN-LONG4-0004

## Discussion

Results of this mixed-methods study point to two major conclusions concerning AMU and related practises among Maasai pastoralists in northern Tanzania. First, the self-reported observance of prudent AMU and AMR-related practises are limited. These results are not unexpected given that such practises appear to be widespread in agricultural communities throughout LMICs (6–13, 38) and are concerning given the links between AMU and the emergence and transmission of AMR (40). Second, there is a disconnect between the KAP concerning prudent AMU and AMR. That is, those who hold prudent use knowledge and attitudes were generally not more likely to observe prudent practises and *vice versa*. This second conclusion is also consistent with results from KAP analyses in Africa (6) and Asia (9, 15). However, we expand upon these previous studies through qualitative inquiry that, as discussed further below, demonstrated the diversity of cultural and historical reasons motivating the lack of significant correlation between KAP. Below, we highlight three of the reasons that often interact to pattern AMU and AMR related practises including: (1) Maasai self-perceptions as veterinary experts, (2) the central role of livestock, particularly cattle, in Maasai culture, and (3) the use of ethnoveterinary knowledge in the patterning of animal health treatment.

Our previous work among the Maasai has highlighted how their self-perception as “veterinary experts” limits their seeking of advice from animal health professionals and impacts their confidence in these professionals (14). As with health-seeking practises, we observe the impacts of these self-perceptions on antimicrobial administration practises. Indeed, the “correctness” of administration practises is not primarily determined through the biomedical expertise of a veterinarian or by referring to instructions on the bottle or sachet. Instead, ethnomedical beliefs, including that that healthier livestock can “withstand” higher doses than weaker animals patterned administration practises. These beliefs may encourage the overdosing of healthy animals and underdosing of sick animals and in turn increase selection pressures for AMR (4). While these practises could be addressed by input from animal health professionals, Maasai self-perceptions as experts combined with the limited accessibility of these professionals in rural areas inhabited by the Maasai mean that such corrections are unlikely (14).

Interestingly, we found that those Maasai who agreed that medicines used inappropriately limited drug effectiveness were *less likely* to follow instructions from veterinarians. This pattern might be expected if Maasai believe that the biomedical advice provided by veterinarians was inferior to their own ethnomedical knowledge, thereby increasing the likelihood that drugs were used inappropriately if advice from these professionals was heeded. Likewise, self-perceptions as veterinary experts likely contributed to the small number of Maasai (i.e., ≈6%) who reported to “sometimes” or “always” get prescriptions for antimicrobials. Prescriptions may be perceived as an unnecessary cost if information from the veterinarian is not perceived as more correct.

Finally, the central role of livestock in the Maasai culture served as a backdrop for rationalising their animal health practises. Livestock, particularly cattle, are perceived as gifts from God and, as such, are bestowed with a sense of purity. Wasting the products provided by these gifts (meat and milk) violates social norms among the Maasai (41–43). Given the continuity of the Maasai culture is based upon the continuity of their herds, throwing away products is a symbolic act that challenges cultural survival. The behavioural consequences of these beliefs are clear in withdrawal practises with very few Maasai (≈5%) reporting that they threw away products during the withdrawal period. Rationales for continued consumption focused on the purity of the God-given livestock products, which meant the Maasai do not have to “fear” these products. Ideas of purity are further supported by historical considerations, with many Maasai saying that they had never seen anyone getting sick from animal products.

### Future Studies and Lessons Learned

This mixed-methods study provides important insights into future investigations of AMU and AMR-related KAP as well as efforts to design AMR awareness campaigns upon these investigations. First, survey instruments that quantify knowledge and attitudes solely based upon “Western” biomedical understandings of antimicrobials and AMR are unlikely to identify the myriad of social, cultural, and historical reasons patterning AMU practises. As this study demonstrates, some of these reasons, including the role of livestock in cultural continuity, are likely to account for a considerable amount of behavioural variance within a community. These reasons, as our results suggest, may supersede any knowledge of the biomedical realities of AMR. Even when this biomedical knowledge is significantly related practises, as we documented with knowledge of withdrawal, the impact on behaviour may be limited. For example, only about 26% of Maasai who reported knowing about withdrawal also reported observing withdrawal. Acknowledging these discrepancies is vitally important in efforts to limit AMR as awareness campaigns and other behavioural change interventions are often based on the KAP study results. These interventions, by not assessing the broader structural issues underlying animal health practises, risk creating better-informed communities that are still engaging in the same non-prudent antimicrobial practises.

## Data Availability Statement

The original contributions presented in the study are included in the article/Supplementary Material, further inquiries can be directed to the corresponding author/s.

## Ethics Statement

The study was reviewed and approved by Medical Research Coordinating Committee of the National Institute for Medical Research (NIMR) in Tanzania (clearance no. NIMR/HQ/R.8a/Vol.IX/2926). Written informed consent was not provided because illiteracy among the Maasai community means written consent could not be secured from all participants. If written consent could be not secured, a thumbprint was secured.

## Author Contributions

PM, MC, EM, MO-N, AD-G, and EK conceived the study and developed the methodology. PM, MC, EM, and MO-N conducted the fieldwork. PM and MC conducted formal analysis. PM and MC prepared the original draught. EM, MO-N, TK, AD-G, and EM reviewed and edited the manuscript. All authors have read and agreed to the published version of the manuscript.

## Conflict of Interest

The authors declare that the research was conducted in the absence of any commercial or financial relationships that could be construed as a potential conflict of interest.
