# Supplementary material for: Knowing Is Not Enough: A Mixed-Methods Study of Antimicrobial Resistance Knowledge, Attitudes, and Practises Among Maasai Pastoralists
Source: Front Vet Sci. 2021 Mar 22;8:645851. doi: 10.3389/fvets.2021.645851 (PMC8023390; doi:10.3389/fvets.2021.645851)
Supplement: Supplementary file 1 [file Data_Sheet_1.docx]

Supplement

Knowing is not enough: A mixed-methods study of antimicrobial resistance knowledge, attitudes, and practices among Maasai pastoralists.

Peter Mangesho^1*^ ^†^, Mark A Caudell^2†^, Elibariki Mwakapeje^3^, Moses OleNesselle^3^, Tabitha Kimani^2^, Alejandro Dorado-Garcia^4^, Emmanuel Kabaili^4^, and Folorunso O. Fasina^3^

**1. Study Locations**

Orbomba ward is where the Longido District headquarters are located and the main road from Arusha City to Namanga Border (between Tanzania and Kenya) runs through the ward. Longido District is also the center of most businesses in the district and offers reliable transportation to the urban center of Arusha. Sinya is located 30 kms from Longido District headquarters and overlooks the slopes of Mount Kilimanjaro and is also adjacent to a wildlife management area. Gelailumbwa is the furthest of all wards (55 kilometers) from Longido District headquarters bordering Ngorongoro District to the northeast. Very little crop cultivation takes place in this area because of it being at the interface of wildlife conservation. This is almost also the same with Sinya ward where the village is part of a community wildlife management area consortium, meaning wildlife freely roams inside it. This ensures that pastoralists depend heavily on their livestock in Sinya Maasai in Engarinaibor rely on a more diverse set of economic activities. There is a greater reliance on crop cultivation while the ward is also home to a large ruby mine. In addition, there are some differences in educational attainment across the four wards. These differences could be attributed to distances to the main road. Orbomba is near the main road and it is thereby easier for district officers to manage/ follow up with Maasai children from Orbomba who may be missing school. In contrast, Gelailumbwa and Sinya are farther away from districts offices and so enforcement of school attendance may be less strict.

**2. Sampling**

Focus group discussion participants were selected from different villages forming the wards. They were purposefully identified by village elders (through respective village hamlet leaders) ensuring that each hamlet (hamlets form villages) was represented in the discussion. Due to resource limitations, a purposive sampling strategy was used for KAP interviews and employed two selection frameworks. First, given the large distances between Maasai bomas, Maasai men and women were interviewed at four livestock markets within the wards (i.e., Orbomba, Engarenaibor, Sinya, and Gelailumbwa). However, the length of the survey dissuaded some individuals from participating given they were preparing to sell or buy items at the market. The second selection framework was interviewing Maasai household heads within their bomas. Households were targeted using village hamlet leaders passing out information to willing interviewees and also a chain-referral method with referrals being generated by informants and research team members (Biernacki and Waldorf, 1981). Within markets and households, enumerators targeted those individuals who were knowledgeable and willing to talk about veterinary drug use, purchasing patterns, and livestock management practices. Most ended up being the household heads, hence our sample (N=195) was biased towards men. KAP surveys were administered by six local enumerators using tablets with the Kobo Collect® application. Enumerators were trained in both qualitative and quantitative methods over a two-week period. Each survey took around 1 hour to complete. All enumerators were Maasai except for one individual. The non-Maasai enumerator, however, had over 5 years’ experience conducting surveys within Maasai communities with first author PM.

Importantly, we did not employ a random sampling strategy and did not achieve sample sizes sufficient to reach representative samples for our targeted geographical areas. As such, results regarding Maasai demographics, socioeconomics, and knowledge, attitudes, and practices should be considered as conclusions relevant to Maasai surveyed in this study.

**Table S1. Number of focus group discussions (FGDs) conducted per ward.**

| **Ward Name** | **Village name** | **Male** | **Female** |
| --- | --- | --- | --- |
| Orbomba | Orbomba | 1 | - |
| Sinya | Ildonyo | 1 | 1 |
| Gelailumbwa | Gelailumbwa | 1 | - |
| Engarinaibor | Mairowa | 1 | 1 |
| Total |  | 4 | 2 |

**Table S2. Demographic information of Maasai focus group discussion (FGD) participants by ward. Wards are in the first row with totals across wards in the final column.**

|  | **GELAILUMBWA** | | **ENGARENAIBOR** | | **ORBOMBA** | | **SINYA** | | **Total** | |
| --- | --- | --- | --- | --- | --- | --- | --- | --- | --- | --- |
|  | N | % | N | % | N | % | N | % | N | % |
| **Sex** |  |  |  |  |  |  |  |  |  |  |
| Female | 0 | 0 | 13 | 81.25 | 0 | 0 | 9 | 52.94 | 22 | 40.00 |
| Male | 12 | 100 | 3 | 18.75 | 10 | 100 | 8 | 47.06 | 33 | 60.00 |
| **Education level** |  |  |  |  |  |  |  |  |  |  |
| No education | 3 | 25.00 | 12 | 75.00 | 1 | 10 | 11 | 64.71 | 27 | 49.09 |
| Primary | 8 | 66.67 | 4 | 25.00 | 9 | 90 | 4 | 23.53 | 25 | 45.45 |
| Secondary | 1 | 8.33 | 0 | 0 | 0 | 0 | 2 | 11.76 | 3 | 5.45 |
| **Age-group** |  |  |  |  |  |  |  |  |  |  |
| 20 - 40 | 2 | 16.67 | 12 | 75.00 | 3 | 30 | 9 | 52.94 | 26 | 47.27 |
| 41 - 70 | 10 | 83.33 | 4 | 25.00 | 7 | 70 | 8 | 47.06 | 29 | 52.73 |
| **Marital status** |  |  |  |  |  |  |  |  |  |  |
| Married | 12 | 100 | 11 | 68.75 | 10 | 100 | 13 | 76.47 | 46 | 83.64 |
| Single | 0 | 0 | 2 | 12.50 | 0 | 0 | 2 | 11.76 | 4 | 7.27 |
| Widow | 0 | 0 | 3 | 18.75 | 0 | 0 | 2 | 11.76 | 5 | 9.09 |

**Table S3. Fisher’s exact test results.** Significant associations are in bold.

|  | Treatment  Period | Group Treat | Overdose | Underdose | Prescription | Observe  Withdrawal |
| --- | --- | --- | --- | --- | --- | --- |
| Can explain AMR | 0.356 | 0.657 | 0.442 | **0.009** | 0.117 | 0.189 |
| Agreement: Can stop giving an animal a full course if  symptoms are improving. | 0.077 | - | - | - | - | - |
| If medicines are given incorrectly, they can stop  working | **0.012** | **0.001** | 0.614 | 0.104 |  |  |
| AMs are to prevent group sick | - | 0.623 | - | - | - | - |
| Giving health animals AMS will prevent sickness | - | 0.168 | - | - | - | - |
| Antimicrobials can help healthy animals grow faster and stronger ( | - | - | 0.623 | - | - | - |
| An animal health professional should be consulted before giving treatment | - | - | - | - | 0.196 | - |
| Agree withdrawal | - | - | - | - | - | 0.097 |
| Been told of withdrawal | - | - | - | - | - | **0.0001** |
| Been told of residues | - | - | - | - | - | 0.484 |

**3. Qualitative themes and tool development**

FGDs were concentrated around twelve major themes. The 12 themes related to antimicrobial use (AMU) and antimicrobial resistance (AMR) and included farm management and economic practices, area disease histories, and knowledge, attitudes, and practices relating to AMU and AMR, including use, governance, regulations, policies, and enforcement. The data collection guides were pre-tested and piloted on livestock production systems in Siha District, Kilimanjaro region, on a site resembling proposed Longido pastoral communities between 19 and 21 November 2018**.** Before the pilot, the research team familiarized themselves with the topic guides and ensured all prompts were translated accordingly. After each field pilot day, the research team re-convened and modified the prompts accordingly. The pilot period also provided an opportunity to further improve the qualitative interviewing skills of the recruited research assistants.

Following transcription and translations of the interviews, the translated transcripts were uploaded into Nvivo Version 8 for organization and coding by PM and supported by MN. Themes were developed from an initial pre-determined theme named Livestock diseases from which most results presented in this paper emanated. This was followed by prompts about “health seeking behaviour”. Sub themes emerged following prompts from the interview guides. In each sub-theme, corresponding responses were categorized as “lower codes” to the sub-themes, that is, responses that elaborated and enriched the coded sub theme. See Table SB1 for a summary of theme development and Table SB2 and Table SB3 for interview guides for focus group discussions and in-depth interviews with the themes assessed and associated probes.

**Table S4. Summary of theme development.**

| **Livestock Diseases care seeking** | |
| --- | --- |
| **Sub-themes** | **Lower level codes** |
| Symptom identification & recognition | Starts with family members at the household level |
|  | Disease syndrome confirmation: by head of household |
|  | Disease recognition support from age set members and neighbours |
| Household level veterinary Drug option deliberations | Antimicrobial hoarding: Drug sourced from the house |
|  | Antimicrobial sourced from neighbours |
|  | Antimicrobial sourced from drugstores |
|  | Discussed and agreed by the head of household |
| Antimicrobial application responsibilities | Head of household (if available) |
|  | Eldest age member of household |
|  | Wife/ mother |
| Drug workings observation & confirmation of healing | Normally - 1 -2 days |
|  | Symptoms subsiding observed (e.g. Cough stops, change in hair lustre) |
|  | Animal ability to walk, eat pasture, drink water |
|  | Confirm capabilities and other family learns |
|  | Drug container kept for future reference |
| Case of Drug Failure/ did not work 1 | Neighbors or friends suggest alternative that worked |
| Drug Failure/ did not work-2 | Animal health profession is consulted |
|  | Agrovet or Veterinary doctor (consulted for a better drug only) |
|  | Veterinary consulted for investigation and drug application |
|  | If all of above fails the animal can also be sold to the market |
| Drug failure 3 | Animal is slaughtered and consumed (household and neighbours) |

**Table S5. Focus group discussion interview guide.** Themes/Domains were developed during a workshop in Nairobi (August, 2018), where experts in antimicrobial resistance generated a list of themes/domains impacting antimicrobial use and antimicrobial resistance and probes to assess these themes/domains.

| **Themes/Domain** | **Topic and Probes** *(in italics)* |
| --- | --- |
| Economics / Masuala ya uchumi | 1. What livelihood activities does your community engage in?  - *What would you consider to be the five-priority livelihood activities based on contribution to household income? List from the most significant to the least* - *Can you estimate the percentage livestock income compared to other enterprise in a year?* - *During the year, when does the income from livestock profitable/ better compared to other enterprise?* - *What are the factors influence livestock process (per specie)? How do you ensure maximum profit?* |
| Management practices | 1. We would like to hear a bit about your animals that you keep.  - *What types do you keep and for what purposes?* - *How do you access resources (water & pasture), paying attention to seasonality?*   - *Sources of each resources and challenges faced including diseases*  1. Do you keep records of your farm management in general? If yes, how? If not why?  - *Probe about productivity, sales, purchases, movement of animals, treatments* - *Probe for the responsibilities of each focusing on gendered roles, in case of the Maasai- age set obligations, clan and household (boma) arrangements* |
| Environmental context / Mazingira | 1. Can you tell me how the environment you live in climate and weather conditions influence your farming practices? What impact do they have?  - *Probe for comparison on changes for the last five years* - *Do you experience interaction with wildlife while grazing, and how has this affect your farming?* - *Compared to other pastoral areas is the geographical position favourable or less favourable for livestock keeping?* - *In relation to population increase and livestock increase how have the farming practices been affected? With a focus of interaction with other livestock* - *Would you say living closely with other farmers or apart increase the chance of cross infections?* |
| Livestock Diseases | 1. Now we are going to talk about health problems that occur in our area, affecting livestock.  - *Can you tell me about the common diseases that your animals faced in the last year? List these per species.*    - *For each specie, lets rank from the most significant to the least.*   - *Where possible, discuss the top three, per season*   - *For the top three what were the common symptoms?*  1. For these diseases that you described above, can you tell me where they came from and how the animals were affected? 2. Health seeking practices  - *For each disease/ health condition what did you do?* - Probe on disease identification - *Probe on the treatment options taken* - *Where did you seek help and why?*  1. What is your preferred source of animal health information and why?  - *Probe on all possible angles, shops, friends, family, healers, drug peddlers, officials* - *What is your preferred source of medicines for treating livestock and why? Give examples of each* |
| AMU | 1. In your day-to-day life do you talk of diseases caused by different kinds of parasites, such as bacteria’s and viruses? (probe on local generally disease causality understanding)  - Can you give examples of the different disease causative agents that you know?  1. In the process of identifying drugs for treating your livestock do you differentiate between antimicrobials and non-antimicrobials?  - How do you do that? - If not, how do you categorize drugs for treatment - *Do the options you take always work for you? Give experiences in the past year as far as you can remember* - Can you name the drugs and the possible diseases they can treat? Please explain how *and the exact measurements as per their understanding* - *Prompt specifically on the different places these are obtained* - *Ask for his opinion on these different sources focusing on issues of quality* - *Ask what strategies they use to decide from which source and why? Pay attention to elaborate on differentiating their own preferences and that of farmers* - *Prompt for experiences in carrying or storing practices by the farmers, and reasons for this*  1. Is there any difference between vaccines and other drugs including antimicrobials?  - *Can you give me examples of vaccines and when do you use them vaccine (ask for names with examples)* - *Where do you and farmers source antimicrobials for use in your community?*  1. In your farming practices, when do you normally administer antimicrobials? Probe for triggering actions such as    1. *Whether during prophylaxis, and why?*    2. *When an animal is sick with symptoms,*    3. *If the biosecurity and hygiene conditions are a course for concern* 2. *If time allows probe for reasons behind each use: whether all round costs, simply hygienic reasons, accessibility affecting quality etc* 3. How do you decide how much antibiotic (dose) to give to your animals, and for how long (treatment course) and how often?  - *Can you always get the kinds of antimicrobials you want to use? Why / why not?* |
| AMR | 1. Have you experience a situation whereby a drug is not working?  - *If yes, please give vivid examples and names of these drugs and what you intended to treat with?* - *How do you tell when a drug or the combination is not working?* - *When this happened what did you do?* - *Probe for all the options taken and why* - *If a combination of drugs was used before or after treatment failure ask for the names of the drugs and the amount of dose given to the animal?*  1. When this drug(s) do not work well as you explain before, how do you call this in your local terms or day-to-day farming practices?      - *Probe for any local or vernacular language, or if informed by the local service provider.* - *What do you think contributes/causes the drug not work properly to the extent you resort to other options? Give examples*  1. When you increase dosage of a drug what happens in the body of the animal?  - *To your understanding what is the meaning of drug volume or CC?* - *What drugs do you normally start with? What other drugs are given next. Give examples please as per disease* - *Can you explain how the following factors influence prescription and use of AMs? Costs of AMs,*   Withdrawal   1. After you treat your livestock and the treatment is not working  - *If you decide to slaughter and consume the meat when can you do that? What treatment is okay to use in the animal and slaughter the same day?*  1. *If you treat a livestock that you are milking what happens with the milk? (Please provide an example and the name of the drug)*  - *How do others in your community behave regarding withrdrawal? (discuss local context meanings and understandings of withdrawal?*  1. Do you normally talk among farmers (or animal health officers) about the relationship between drugs used in animals and whether they can get into humans?  - *If yes, what is the connection and how do you describe it in local lingo?* - *If they do get into humans what happens in the human body? What do you think is the outcome and why?* - *If no, do you think the drugs that are treated on livestock can get to humans through milk or meat? What do you think happens?*  1. If antimicrobials stopped working, what would that mean for you and your farm? |
| Summary and any other questions | *(Summarise the main points of the participant checking their agreement and asking if they have anything more to add).*  Are there any points you think I’ve missed, or any other issues you’d like to talk about relating to AMU and AMR? |

**References**

Biernacki, P., Waldorf, D., 1981. Snowball sampling: Problems and techniques of chain referral sampling. Sociological methods & research 10, 141–163.
